# Risk factors and prediction model of dysphagia in patients with recent small subcortical infarct

**DOI:** 10.1007/s10072-025-08648-z

**Published:** 2026-01-10

**Authors:** Yizhen Weng, Jie Li, Wenxiu Li, Hui Guo, Xinyi He, Qi Fang, Xiang Tang, Lulu Zhang

**Affiliations:** 1 Department of Neurology, First Affiliated Hospital of Soochow University, No. 899, Pinghai Road, Suzhou, 215006 Jiangsu China; 2 Department of Rehabilitation Medicine, Liyang People’s Hospital, No. 70, Jianshe West Road, Liyang, 213300 Jiangsu China

**Keywords:** Dysphagia, Recent small subcortical infarct, Cerebral small vessel disease, Nomogram

## Abstract

**Background:**

The study aims to determine the association of risk factors and other major imaging markers of cerebral small vessel disease (CSVD) with post-stroke dysphagia (PSD) in patients with recent small subcortical infarct (RSSI), establish a predictive model and evaluate its predictive effectiveness.

**Methods:**

A total of 394 patients with RSSI were enrolled in this study, with 79 (20.05%) of them diagnosed with PSD. Swallowing function assessments, including the water-swallowing test(WST) and volume-viscosity swallow test (V-VST), were conducted within the first 24 h following admission for oral feeding. Demographic and clinical data were collected from our stroke database. Major imaging markers of CSVD were evaluated through MRI scans. Multivariate logistic regression analysis was employed to identify independent risk factors for PSD in RSSI patients. Subsequently, a nomogram involving all these independent risk factors was developed and validated by Bootstrap. Receiver Operating Characteristic (ROC) curve analysis, calibration curve analysis, and decision curve analysis (DCA) were used to assess the predictive performance of the model.

**Results:**

Imaging markers of cerebral small vessel disease, including cerebral microbleeds (CMBs) (OR = 3.939, 95% CI: 1.613–9.616), and moderate to severe enlargement of perivascular spaces (EPVS) (OR = 2.276, 95% CI: 1.160–4.466), were found to be significantly associated with PSD in patients with RSSI. The risk factors related to dysphagia in patients with RSSI included the following: High-sensitivity C-reactive protein (hs-CRP) (OR = 1.076, 95% CI: 1.005–1.153),baseline National Institutes of Health Stroke Scale (NIHSS) score (OR = 1.230, 95% CI: 1.132–1.336), total CSVD burden (OR = 1.613, 95% CI: 1.195–2.177) and lesion region(OR = 4.462, 95%CI: 2.333–8.532). The nomogram based on the four independent risk factors was developed and validated by Bootstrap. The model demonstrated an excellent predictive performance, with an area under the receiver operating characteristic curve (AUC) of 84.7%. The calibration curve indicated that the model's predictions closely align with actual outcomes, and DCA confirmed the model's clinical utility.

**Conclusion:**

CSVD imaging markers, such as CMBs and moderate to severe EPVS, are associated with PSD in RSSI patients. Key risk factors were identified, and a predictive model was developed, which could serve as an effective tool for assessing individual risk and optimizing clinical decision-making in RSSI patients.

## Introduction

Dysphagia, characterized by impaired ability to safely and effectively transport food from the mouth to the stomach, may result from a variety of conditions, including neurodegenerative disorders, head and neck tumors, with stroke being the most common cause [1]. Recent epidemiological studies have shown that more than 50% of acute stroke patients experience dysphagia [2]. Post-stroke dysphagia (PSD) can lead to serious complications, such as aspiration pneumonia, malnutrition, prolonged hospitalization, and increased medical expenditures [1, 2]. These complications significantly hinder rehabilitation, contributing to poor prognosis, as well as higher morbidity and mortality rates among stroke patients [2, 3].

It is noteworthy that nearly one in five individuals with recent small subcortical infarct (RSSI) experience PSD [4, 5], underscoring the importance of early identification and intervention to prevent serious complications. RSSI is considered an acute manifestation of cerebral small vessel disease (CSVD) and is typically characterized by imaging features or clinical symptoms indicative of cerebral injury occurring within the preceding weeks [6]. RSSI accounts for approximately 25% of all ischemic strokes and most commonly involves regions such as the semioval centers, corona radiata, basal ganglia, internal capsule, thalamus, and brainstem [7].

Nevertheless, the exact mechanisms underlying the development of PSD following RSSI remain poorly understood and have not been thoroughly validated. RSSI is regarded as an acute manifestation of CSVD and is often accompanied by other CSVD-related imaging markers, such as white matter hyperintensities (WMHs), cerebral microbleeds (CMBs), lacunes, and enlarged perivascular spaces (EPVS) [8]. It is common for patients to exhibit two or more CSVD imaging features simultaneously. Previous studies have identified several risk factors for PSD in RSSI patients, including greater stroke severity, extensive WMHs, and pontine infarction, while no significant associations have been found with CMBs or lacunar infarcts [4]. However, contrasting evidence suggests that severe WMHs may not be associated with dysphagia in patients with supratentorial RSSI, but rather that the anatomical location of lacunar infarcts or white matter lesions may be more relevant [5]. Moreover, the total imaging burden of CSVD has been shown to independently contribute to the risk of PSD in this population [9]. These findings underscore the complexity of PSD pathogenesis in the context of RSSI and highlight the need for further investigation into the relationship between CSVD imaging markers and PSD occurrence.

Current guidelines for dysphagia recommend that all acute stroke patients undergo routine screening for swallowing function before eating [10]. Additional swallowing assessments should be performed if there is a high suspicion or risk of dysphagia. This study utilized the Water Swallowing Test (WST) combined with the Volume–Viscosity Swallow Test (V-VST) to assess swallowing function within 24 h and promptly identify PSD patients. However, clinical practice reveals that only 36.52% of caregivers perform early screening as recommended by the guidelines [11]. Currently, there is no internationally recognized or standardized approach to dysphagia screening. Common screening tools include the 3-oz Water Swallow Test, the Acute Stroke Dysphagia Screen, the Toronto Bedside Swallowing Screening Test (TOR-BSST), and the Burke Dysphagia Screening Test. Despite their widespread use, these tools may fail to detect 2%–50% of dysphagic patients [12], particularly those without obvious clinical symptoms such as aspiration, coughing, or voice changes [13]. While bedside evaluations and instrumental assessments, such as videofluoroscopic swallowing studies or fiberoptic endoscopic evaluations, offer greater diagnostic accuracy, their use in routine clinical practice is limited by technical complexity, the need for specialized personnel, and prolonged waiting times [14].

In recent years, researchers have developed risk prediction models for dysphagia [15–18]. For instance, recent studies have focused on predicting dysphagia in ICU patients post-extubation, aiming to identify high-risk individuals and facilitate early personalized interventions [18]. Similarly, prediction models for dysphagia following radiotherapy have been established internationally [17]. However, these models were developed for specific populations and are not applicable to RSSI patients.

Therefore, this study leverages comprehensive clinical data from early clinical swallowing assessments and magnetic resonance imaging (MRI) markers of CSVD to explore the risk factors for dysphagia in RSSI and their correlation with CSVD imaging markers. The aim is to develop an economical and simple predictive model to identify high-risk dysphagia patients, thus facilitating early, individualized interventions and reducing the complications associated with dysphagia.

## Materials and methods

### Participants

Participants were recruited from patients diagnosed with RSSI who were admitted to the First Affiliated Hospital of Soochow University between October 2017 and January 2022. The inclusion criteria were as follows: (1) diagnosis of RSSI confirmed by MRI, with lesions located in the supratentorial regions (including the internal capsule, corona radiata, basal ganglia, and thalamus) or the brainstem; (2) patients experiencing their first cerebral infarction within 14 days of onset [4, 19]; (3) patients who underwent head MRI following the cerebrovascular accident; (4) patients able to cooperate with the assessment and evaluation; and (5) provision of informed consent by the patient or their legal representative. Exclusion criteria included: (1) multiple acute subcortical infarcts or additional infarcts at other locations; (2) patients with conditions that could affect swallowing function, such as esophageal cancer; (3) patients with unstable vital signs; (4) patients with mental, cognitive, or speech disorders who were unable to cooperate with the evaluator; (5) patients with preexisting dysphagia; and (6) patients who underwent emergency endovascular treatment after stroke onset.

Participants were subsequently categorized into two groups based on swallowing function assessments: (1) patients with PSD and (2) patients without PSD.

### Clinical data

Upon admission, demographic and clinical data were collected, including age, sex, baseline systolic and diastolic blood pressure, blood glucose levels, and lipid profile parameters (triglycerides, total cholesterol, and low-density lipoprotein cholesterol). Additional laboratory indicators relevant to the study were also recorded. Medical history was obtained, covering comorbidities such as hypertension, diabetes mellitus, prior stroke, and smoking status. Stroke severity at baseline was evaluated using the NIHSS.

### Swallowing assessment

Swallowing ability was evaluated within 24 h of admission by a neurologist and a nurse, both blinded to the clinical data. Assessment was conducted using two standardized tools [20]: WST and V-VST. For the WST, patients were seated upright at a 90° angle and instructed to drink 30 mL of water. Swallowing patterns were classified into five categories: (1a) single swallow without choking within 5 s; (1b) single swallow without choking taking more than 5 s; (2) multiple swallows without choking; (3) single swallow with choking; (4) multiple swallows with choking; and (5) choking with inability to complete the 30 mL intake. Patients scoring ≥ 1b proceeded to the V-VST for further evaluation.

The V-VST involved administering boluses of varying volumes (5, 10, and 20 mL) and viscosities (thin liquid, nectar-like, and spoon-thick), along with continuous pulse oximetry monitoring to assess swallowing safety and efficacy. Indicators of impaired safety included coughing, voice changes (e.g., wet or gurgly voice), or a ≥ 5% drop in oxygen saturation (SpO₂) from baseline. Impaired efficacy was defined by signs such as oral or pharyngeal residue, spillage, or incomplete swallowing. A diagnosis of dysphagia was made if any impairment in safety or efficacy was observed during the V-VST.

### MRI analysis

Magnetic Resonance Imaging (MRI) was conducted within three days of admission using a 3.0 T MRI scanner. The imaging sequences included T1-weighted imaging (T1WI), T2-weighted imaging (T2WI), Diffusion-weighted imaging (DWI), Fluid-attenuated inversion recovery (FLAIR), and Susceptibility-weighted imaging (SWI). All images underwent blinded assessment by both a neurology expert and an imaging specialist. In cases of disagreement, consensus was reached through discussion.

According to the Consensus on the Diagnosis and Treatment of CSVD [21], RSSI are defined as small infarctions within the perforating artery territory, measuring < 20 mm in axial diameter and > 20 mm on coronal or sagittal images. Based on lesion location, RSSI are classified into supratentorial regions (centrum semiovale, corona radiata, basal ganglia, thalamus) and the brainstem. WMHs are abnormal signal areas within the white matter, appearing hypointense or isointense on T1WI and hyperintense on T2WI and FLAIR. The Fazekas [22] scale assesses WMH severity in periventricular (PVWM) and deep white matter (DWM), with total scores ranging from 0 to 6.CMBs are identified on SWI as small, homogeneous, rounded lesions with a diameter of less than 10 mm. Lacunes are defined as circular or oval-shaped hyperintense lesions in subcortical areas with diameters ranging from 3 to 20 mm. They appear hypointense on T1WI and hyperintense on T2WI, with a hyperintense rim on FLAIR and no increased signal on DWI. Enlarged perivascular spaces (EPVS) are small (< 3 mm), punctate lesions when viewed perpendicular to the imaging plane and linear when parallel to it. They appear hypointense on T1WI and FLAIR, and hyperintense on T2WI. According to previous studies [23, 24], EPVS in the frontal cortex, centrum semiovale, basal ganglia, hippocampus, midbrain, and pons were scored separately using the following scale: 0 = none, 1 = 1–10, 2 = 11–20, 3 = 21–40, and 4 = > 41 EPVS. Mild EPVS was defined as grades 0–1, whereas moderate to severe EPVS corresponded to grades 2–4.

### Total CSVD imaging burden score

The total CSVD imaging burden score was determined using a standardized scale [25], assigning one point for each of the following: presence of lacunes; Fazekas score of 2–3 for deep WMHs and/or a score of 3 for periventricular WMHs; presence of CMBs; and moderate to severe (grade 2–4) EPVS. The total score ranges from 0 to 4, with higher scores indicating a greater CSVD imaging burden.

### Statistical analysis

All statistical analyses were performed using SPSS version 26.0. Continuous variables with a normal distribution were presented as mean ± standard deviation (x̄ ± s) and compared using independent sample t-tests. Non-normally distributed continuous variables were expressed as median and interquartile range (IQR) and compared using the Mann–Whitney U test. Categorical variables were presented as frequencies and percentages (%) and analyzed using the chi-square test or Fisher’s exact test, as appropriate.

Univariate analyses were conducted to assess baseline characteristics and cerebral small vessel disease (CSVD) imaging markers. Variables with *p*-values < 0.05 in the univariate analysis were included in a multivariate logistic regression model to control for potential confounders and identify independent risk factors for dysphagia in patients with RSSI. A nomogram prediction model was constructed using R software (version 4.2.1). Model performance was evaluated in terms of discrimination, calibration, and clinical utility. Internal validation was conducted through Bootstrap resampling. A two-sided p-value < 0.05 was considered statistically significant.

## Results

### Baseline characteristics

A total of 394 patients met the inclusion criteria and were enrolled in this study. Among them, 271 (68.78%) were male and 123 (31.22%) were female, with a mean age of 62.01 ± 13.33 years. Of the participants, 79 (20.05%) were diagnosed with PSD. Among the 288 (73.10%) patients with supratentorial small subcortical infarcts and 105 (26.90%) with brainstem RSSI, PSD was observed in 41 (14.24%) and 38 (35.85%) patients, respectively. In the cohort with RSSI, additional comorbid factors were also prevalent: 39 patients (9.9%) had CMBs, 130 patients (32.99%) had moderate to severe EPVS, 217 patients (55.08%) had lacunes, and 289 patients (73.35%) had WMHs.

### Risk factors for dysphagia in patients with RSSI

Univariate comparisons between patients with and without PSD are summarized in Table 1. Patients in the PSD group were significantly older than those without PSD (68.46 ± 1.15 vs. 60.39 ± 13.35 years, *p* < 0.01). A prior history of stroke was more prevalent in the PSD group (26.58% vs. 13.02%, *p* = 0.003). Inflammatory markers, including hs-CRP and fibrinogen, were markedly elevated in PSD patients [hs-CRP: 5.04 (1.72–13.70) vs. 1.61 (0.80–3.79) mg/L, *p* < 0.001; fibrinogen: 3.23 ± 1.13 vs 2.61 ± 0.91 g/L, *p* < 0.001]. Patients with PSD exhibited more severe neurological deficits, as indicated by higher NIHSS scores [6 (3–11) vs. 2 (1–5), *p* < 0.001], and a greater total burden of CSVD [2 (1–3) vs. 1 (0–2), *p* < 0.001]. Moreover, RSSIs were more frequently located in brainstem among PSD patients (48.10% vs. 21.59%, *p* < 0.001). Conversely, patients without PSD had significantly higher levels of triglycerides and uric acid (both *p* < 0.001). There were no significant differences between the two groups in terms of sex, baseline blood pressure, homocysteine (HCY), low-density lipoprotein (LDL), total cholesterol, fasting blood glucose, glycated hemoglobin (HbA1c), serum creatinine, or history of hypertension, diabetes mellitus, atrial fibrillation, smoking, thrombolysis, or lesion laterality.

**Table 1:** Demographic and clinical characteristics of RSSI patients with and without dysphagia

Demographic and clinical data	Dysphagia (*n* = 79)	Controls (*n* = 315)	t/Z/χ^2^	*P*-value
Age(years)	68.46 ± 1.15	60.39 ± 13.35	t = −4.950	< 0.001
Male(*n*,%)	50(63.29)	221(70.16)	χ2 = 1.387	0.239
Systolic Blood Pressure(mmHg)	149.37 ± 25.01	147.74 ± 21.32	t = −0.586	0.558
Diastolic Blood Pressure(mmHg)	80.85 ± 11.81	82.35 ± 13.87	t = 0.883	0.378
History of hypertension(*n*,%)	63(79.75)	235(74.60)	χ2 = 0.907	0.341
History of diabetes(*n*,%)	29(36.71)	95(30.16)	χ2 = 1.256	0.262
History of AF(*n*,%)	4(5.06)	7(2.22)	χ2 = 0.977	0.323
Previous stroke(*n*,%)	21(26.58)	41(13.02)	χ2 = 8.767	0.003
Smoking(*n*,%)	19(24.05)	90(28.57)	χ^2^ = 0.645	0.422
Thrombolysis(*n*,%)	15(18.99)	62(19.68)	χ^2^ = 0.019	0.889
hsCRP(mg/L)	5.04 (1.72–13.70)	1.61 (0.80–3.79)	Z = −5.989	< 0.001
Fibrinogen(g/L)	3.23 ± 1.13	2.61 ± 0.91	t = −5.194	< 0.001
Homocysteine(μmol/L)	11.20 (8.90–13.60) (*n* = 67)	10.30 (8.53–13.53) (*n* = 276)	Z = −1.310	0.190
Triglyceride(mmol/L)	1.34 ± 0.52	1.66 ± 1.33	t = 4.056	< 0.001
Total cholesterol(mmol/L)	4.27 ± 1.01	4.36 ± 1.03	t = 0.697	0.486
LDLC (mmol/L)	2.58 ± 0.90	2.65 ± 0.90	t = 0.618	0.538
Creatinine(μmol/L)	64.00 (55.2–73.70)	65.70 (57.40–79.7)	Z = −1.115	0.265
Uric acid(μmol/L)	288.65 ± 91.86	315.40 ± 92.13	t = 2.309	0.021
Fasting Blood Glucose(mmol/L)	5.53(5.04–6.38)	5.23(4.73–6.39)	Z = −1.838	0.066
Hemoglobin A1c(%)	6.30(5.90–8.30) (*n* = 59)	6.20(5.70–7.30) (*n* = 250)	Z = −1.554	0.120
NIH stroke score	6(3–11)	2(1–5)	Z = −7.070	< 0.001
Total CSVD burden	2(1–3)	1(0–2)	Z = −5.576	< 0.001
Lesion region(*n*,%)				
Supratentorial Region	41(51.90)	247(78.41)	χ^2^ = 22.578	< 0.001
Brainstem Region	38(48.10)	68(21.59)	χ^2^ = 22.578	< 0.001
Left Hemisphere	46(58.23)	161(51.11)	χ^2^ = 1.283	0.257

Normally distributed continuous variables are expressed as mean ± standard deviation (SD) and compared using independent t-tests. Non-normally distributed variables are presented as median (interquartile range, IQR) and compared using the Mann–Whitney U test (Z values reported). Categorical variables are shown as number (%) and analyzed using the chi-square test or Fisher’s exact test

AF refers to atrial fbrillation, hsCRP refers to hypersensitive C-reactive protein, LDLC refers to low density lipoprotein cholesterol, and CSVD refers to cerebral small vascular disease

Variables with *p* < 0.05 in the univariate analysis were subsequently included in the multivariable logistic regression model. As summarized in Table 2, hs-CRP level (OR = 1.076, 95% CI: 1.005–1.153), baseline NIHSS score (OR = 1.230, 95% CI: 1.132–1.336), total CSVD burden (OR = 1.613, 95% CI: 1.195–2.177), and brainstem region (OR = 4.462, 95%CI: 2.333–8.532) emerged as independent predictors of PSD. Other variables were not significantly associated with PSD in the multivariate model.

**Table 2:** Multivariate logistic regression analysis for predicting dysphagia among patients with RSSI

Variables	Odds ratio(95%CI)	*P*-value
Age	1.021 (0.993–1.050)	0.143
Previous stroke	1.442 (0.683–3.047)	0.337
hsCRP	1.076 (1.005–1.153)	0.037
Fibrinogen	1.209 (0.873–1.673)	0.253
Triglyceride	0.588 (0.334–1.033)	0.065
Uric acid	0.999 (0.996–1.003)	0.743
Baseline NIHSS Score	1.230 (1.132–1.336)	< 0.001
Total CSVD burden	1.613 (1.195–2.177)	0.002
Brainstem region	4.462 (2.333–8.532)	< 0.001

hsCRP refers to hypersensitive C-reactive protein, CSVD refers to cerebral small vascular disease

### Correlation between RSSI dysphagia and imaging markers of CSVD

Given that the total CSVD burden encompasses four MRI features—WMHs, lacunae, CMBs, and EPVS—which are intercorrelated, we excluded the total CSVD burden from the analysis. Instead, we examined five individual variables: periventricular WMHs (PVWMHs) score, deep WMHs (DWMHs) score, presence of lacunae, deep and infratentorial CMBs, and moderate to severe EPVS in the basal ganglia. This approach aimed to clarify the specific associations of these CSVD markers with PSD.

As shown in Table 3, significant differences were observed between the PSD and non-PSD groups in lacunae (72.15% vs. 50.79%, *p* = 0.001), CMBs (22.78% vs. 6.67%, *p* < 0.001), moderate to severe EPVS (54.43% vs. 27.62%, *p* < 0.001), PVWMHs score [2 (0–2) vs. 1 (0–2), *p* < 0.001], and DWMHs score [1 (1–2) vs. 1 (0–2), *p* = 0.001].

**Table 3:** MRI features of CSVD for RSSI patients with dysphagia and controls

MRI features of CSVD	Dysphagia (*n* = 79)	Controls (*n* = 315)	χ^2^/Z	*P*-value
Lacunes(*n*,%)	57 (72.15)	160 (50.79)	χ^2^ = 11.645	0.001
CMBs(*n*,%)	18 (22.78)	21 (6.67)	χ^2^ = 18.398	< 0.001
Moderate to severe EPVS (*n*,%)	43 (54.43)	87 (27.62)	χ^2^ = 20.536	< 0.001
PVWMHs score	2 (0–2)	1 (0–2)	Z = −3.823	< 0.001
DWMHs score	1 (1–2)	1 (0–2)	Z = −3.666	0.001

CSVD refers to cerebral small vascular disease, CMBs refers to cerebral microbleeds, EPVS refers to enlarged perivascular spaces, PVWMHs refers to periventricular white matter hyperintensities, DWMHs refers to deep white matter hyperintensities, moderate to severe EPVS corresponds to grades 2–4

After adjustment for potential confounders—including age, previous stroke, hsCRP, fibrinogen, triglycerides, uric acid, baseline NIHSS score, and lesion region—using multivariate logistic regression (Table 4), CMBs (OR = 3.939, 95% CI: 1.613–9.616) and moderate to severe EPVS (OR = 3.939, 95% CI: 1.613–9.616) remained independently associated with PSD. No significant associations were found for DWMHs score, PVWMHs score, or lacunae.

**Table 4:** Multivariate analysis of the correlation between RSSI dysphagia and CSVD imaging markers

CSVD imaging markers	Odds ratio(95%CI)	*P*-value
DWMHs score	0.945 (0.623–1.435)	0.791
PVWMH score	1.061 (0.703–1.601)	0.779
CMBs	3.939 (1.613–9.616)	0.003
Moderate to severe EPVS	2.225 (1.123–4.409)	0.022
Lacunes	1.294 (0.648–2.583)	0.465

Adjusting factors included age, previous stroke, hsCRP, fibrinogen, triglycerides, uric acid, baseline NIHSS score, and lesion region

### Development and validation of the nomogram

**Fig. 1:**
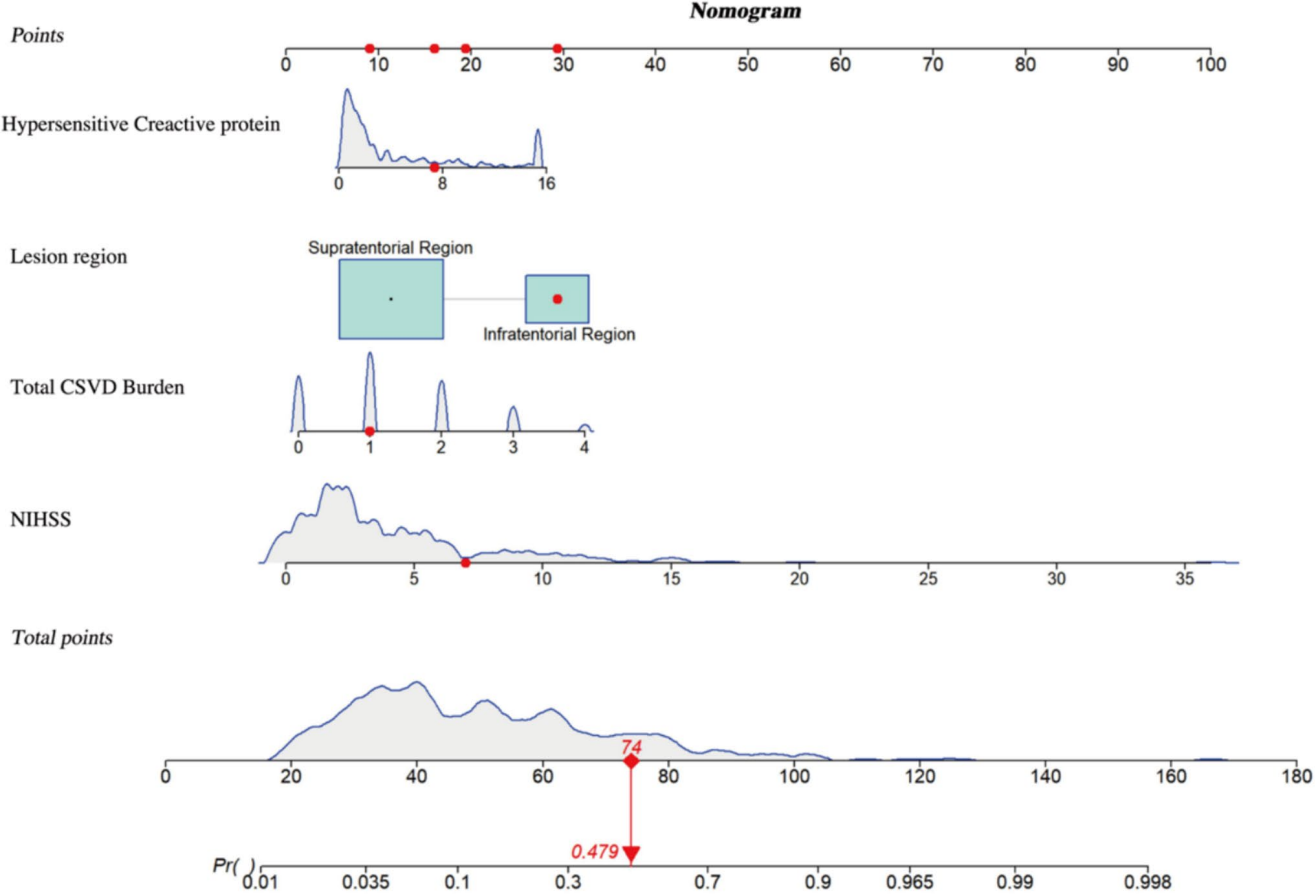
Nomogram predicting the probability of dysphagia in RSSI patients. The nomogram incorporates four independent risk factors: hs-CRP, lesion location, total CSVD burden, and baseline NIHSS score, with blue lines showing the distribution of each variable. As an example, a randomly selected RSSI patient (first row of the dataset) with hs-CRP 7.4 mmol/L, brainstem lesion, CSVD burden 1, and NIHSS 7 has a total nomogram score of 74, corresponding to a predicted dysphagia risk of 47.9%

A nomogram predicting dysphagia risk in RSSI patients was constructed based on multivariate regression results. This nomogram includes four independent predictors: NIHSS score, total CSVD burden, hsCRP levels, and lesion region. A higher total score on the nomogram corresponds to an increased likelihood of developing dysphagia. For instance, consider a patient with RSSI who presents with an hsCRP level of 7.4 mmol/L, a brainstem lesion, a total CSVD burden of 1 point, and a baseline NIHSS score of 7 points. Using the nomogram, the personalized predicted risk score for this patient is 74 points, which corresponds to a dysphagia risk probability of 47.9% (Fig. 1).

**Fig. 2:**
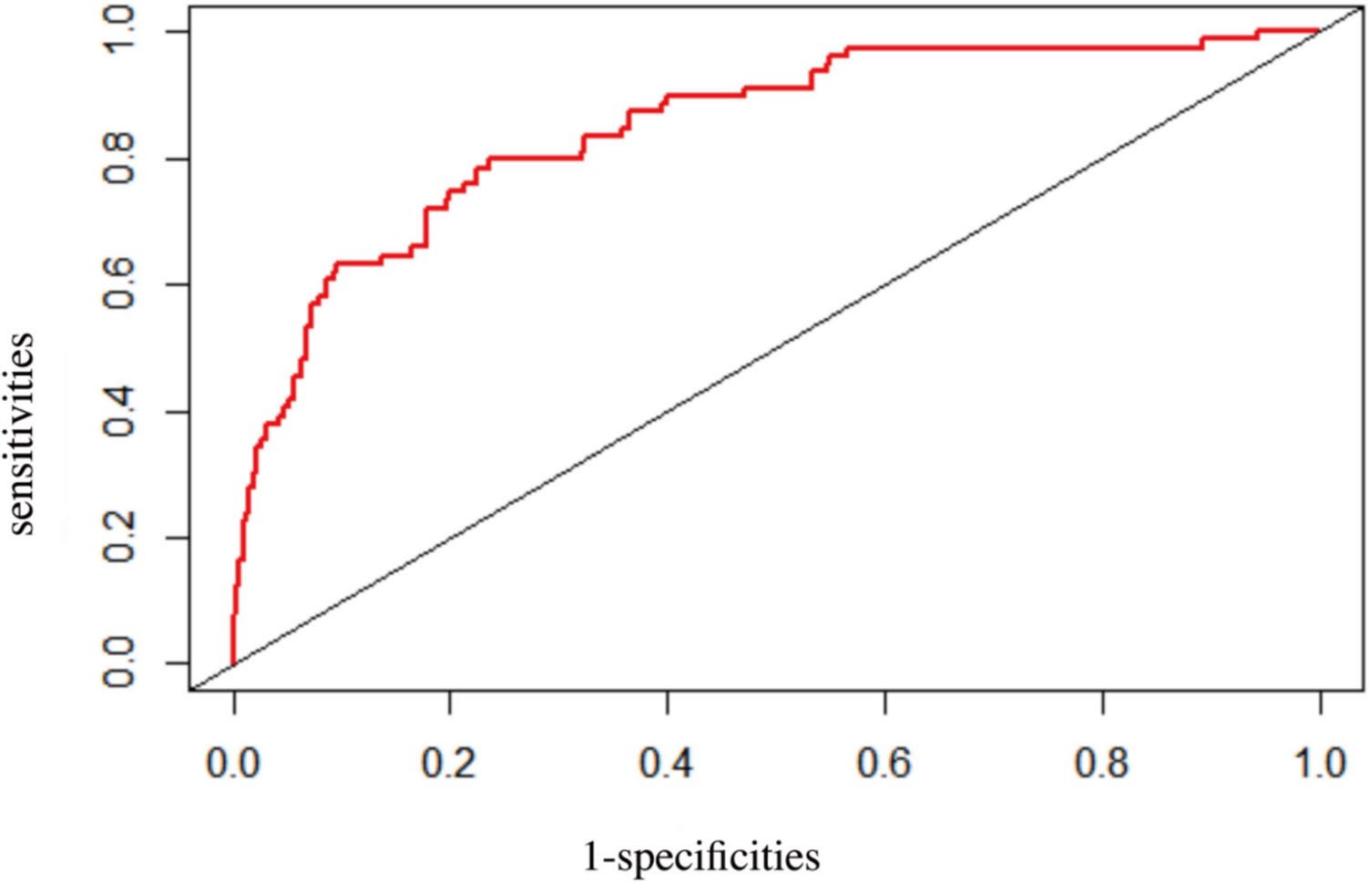
ROC curve of the nomogram for predicting dysphagia risk in RSSI patients

As shown in Fig. 2, our model demonstrated excellent performance with an AUC value of 84.7%. The sensitivity and specificity, determined by the cut-off curve, were 79.7% and 76.5%, respectively. The C-index for the model was 0.847 (95% CI, 0.799–0.896), indicating a high discriminatory ability. The corrected C-index, obtained through bootstrap resampling, was 0.840, further confirming the model's robust internal validation.

**Fig. 3:**
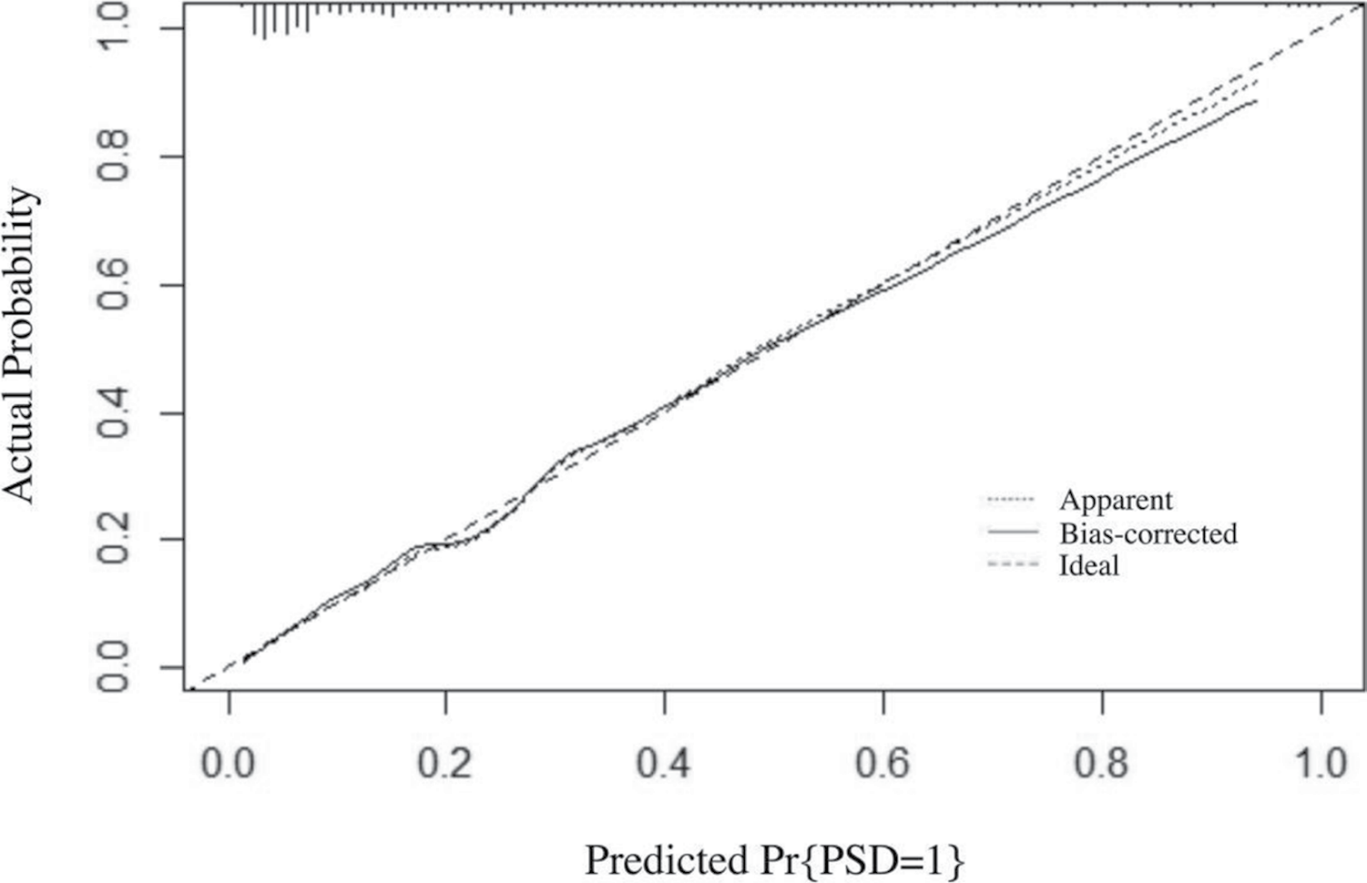
Calibration plot of the nomogram for predicting dysphagia in RSSI patients. The x-axis shows predicted probabilities, and the y-axis shows actual dysphagia rates. The dotted line represents apparent calibration, while the solid line, bias-corrected via 1000 bootstrap resamples (mean absolute error = 0.009), reflects model performance. A perfect prediction aligns with the diagonal dashed line

Figure 3 illustrates the calibration curve based on internal validation with 1000 bootstrap resamples, which reveals a mean absolute error of 0.009. Both the apparent and bias-corrected calibration lines closely align with the ideal line, suggesting that the model's predictions closely match the observed outcomes.

**Fig. 4:**
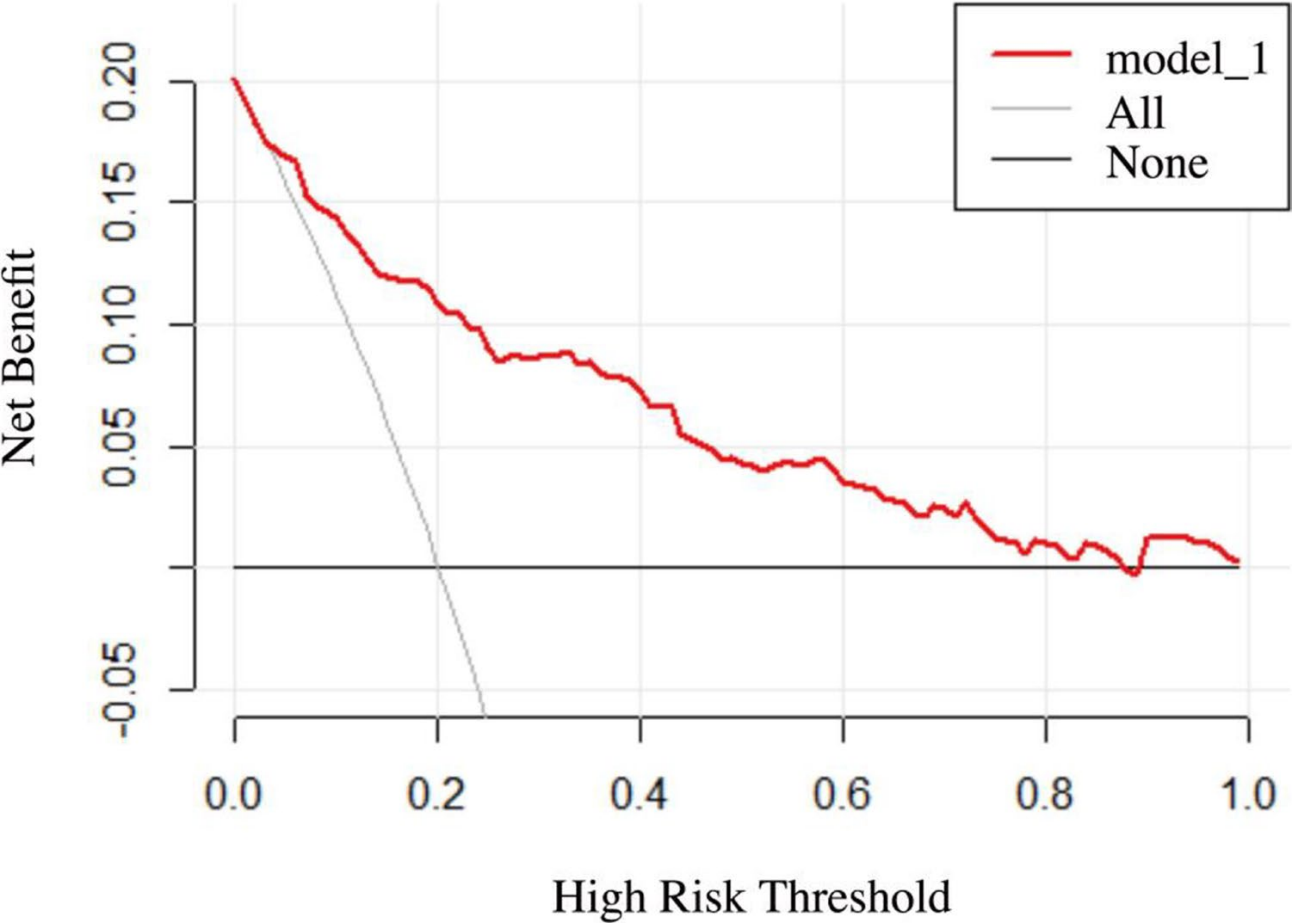
DCA for the nomogram. The y-axis shows net benefit, and the x-axis represents high-risk thresholds (0.05 to 0.9). The black line assumes no dysphagia, with a net benefit of zero. The gray line assumes all patients have dysphagia. The red line represents the net benefit of using model1 (baseline NIHSS score + total CSVD burden + hs-CRP + lesion region) to predict dysphagia in RSSI patients

Additionally, DCA was was conducted to assess the clinical utility of the nomogram. The DCA curves indicate that across threshold probabilities ranging from 0.05 to 0.9, the nomogram-based prediction of dysphagia in RSSI patients provides greater clinical benefit than either a 'treat-all-patients' or 'treat-none' strategy (Fig. 4).

## Discussion

This study presents a reliable and user-friendly nomogram designed to predict the likelihood of PSD in patients suffering from RSSI. The model incorporates four independent and readily measurable risk factors—hs-CRP, baseline NIHSS score, total burden of CSVD, and lesion location. These parameters are easily obtainable in the early phase following RSSI diagnosis, enabling clinicians to promptly identify high-risk patients and initiate appropriate preventive or therapeutic interventions.

Our findings highlight the important role of both clinical and imaging markers in the development of PSD. Notably, NIHSS score emerged as one of the strongest predictors of dysphagia [26–28]. As a well-established indicator of stroke severity, a higher NIHSS score reflects more profound neurological deficits, which are closely associated with impaired motor and sensory functions involved in swallowing. European guidelines already recommend early swallowing assessments in stroke patients with NIHSS ≥ 10 [29], and our study supports this recommendation even in those with moderate stroke severity. In our cohort, the average NIHSS score among dysphagic RSSI patients was 6, underscoring the impact of even moderate neurological impairment on swallowing function.

hs-CRP is a non-specific acute-phase marker of systemic inflammation and a more sensitive indicator than conventional CRP. Previous studies have shown that elevated CRP levels, reflecting systemic inflammation, are closely linked to the onset of dysphagia in patients with RSSI [9].Furthermore, both CRP and hs-CRP have been implicated in the onset and progression of CSVD [30, 31], particularly in relation to key neuroimaging features such as WMHs, lacunar infarcts, CMBs, and EPVS in the basal ganglia. Increased levels of CRP and hs-CRP may aggravate CSVD burden, thereby disrupting the integrity of neural networks involved in swallowing and contributing to a higher risk of dysphagia [32].Our findings provide further evidence that higher hs-CRP levels independently predict dysphagia in individuals with RSSI.

Importantly, our study reinforces the role of CSVD imaging markers in PSD pathogenesis. CMBs and moderate-to-severe EPVS were found to be independently associated with dysphagia, offering insight into the pathophysiological processes linking microvascular pathology with swallowing dysfunction. Patients with brainstem infarctions in our study exhibited the highest prevalence of dysphagia, consistent with the known anatomical location of the swallowing center [33–35]. Mechanistically, CMBs and EPVS may disrupt swallowing networks through complementary pathways. CMBs, often located in subcortical regions, can damage corticobulbar tracts connecting the cerebral cortex to the brainstem, thereby impairing motor control of the pharyngeal and laryngeal muscles, which are critical for safe swallowing [36]. Meanwhile, EPVS, commonly observed in the basal ganglia and subcortical white matter, may interfere with subcortical circuits that modulate cortical input to the brainstem, further compromising swallowing coordination [37]. Together, these microvascular injuries can disrupt the integration of cortical and brainstem networks, ultimately leading to impaired execution of safe and efficient swallowing.

Interestingly, age—a well-established determinant of dysphagia—lost its predictive significance after adjustment. Although age was significantly associated with PSD in the univariate analysis, with patients in the PSD group being notably older than those without PSD, this association disappeared after controlling for confounders, particularly the total CSVD burden score. This finding suggests that the effect of age on PSD risk may be partially mediated through CSVD-related pathology. Age is strongly associated with the development and progression of CSVD, and several studies have demonstrated that increasing age is one of the most robust predictors of greater CSVD burden [38, 39]. Therefore, the loss of significance after adjustment may reflect collinearity between age and CSVD markers or potential over-adjustment, highlighting the intertwined relationship between aging, CSVD pathology, and dysphagia risk.

Moreover, we did not observe significant associations between PSD and other CSVD markers such as periventricular/deep WMHs or lacunes [5]. Although WMHs are anatomically relevant to swallowing function [9, 40, 41], they did not emerge as an independent predictor of PSD in our cohort. Several factors may explain this discrepancy compared with previous studies. First, WMH burden was evaluated using the Fazekas visual scale rather than quantitative volumetric methods. The semi-quantitative and partly subjective nature of this approach may have limited the sensitivity to detect associations. Second, WMHs are strongly correlated with age, which could lead to collinearity and unstable coefficient estimates in multivariate models. Third, the relatively small number of PSD cases in our cohort may have reduced statistical power to identify independent associations. Future studies with larger sample sizes and quantitative volumetric assessments are needed to clarify the role of WMHs in predicting PSD.

Overall, our study addresses a critical gap in existing dysphagia prediction models, which have largely focused on specific populations—such as post-extubation ICU patients or individuals undergoing radiotherapy [17, 18]—and therefore lack direct applicability to the RSSI population. By concentrating on patients with RSSI and integrating both clinical factors and CSVD-related imaging markers, our nomogram provides a more tailored and clinically meaningful tool for early PSD risk stratification. This approach enhances its practical value in routine clinical settings and underscores the importance of considering both neurological impairment and underlying microvascular pathology in the comprehensive assessment of dysphagia risk following subcortical infarction.

Though the results offer valuable insights, it is important to acknowledge several limitations. First, this study was conducted at a single center, which may restrict the generalizability of our findings. Therefore, external validation using larger, multicenter cohorts is warranted to enhance the applicability and robustness of the nomogram. Specifically, we plan to collaborate with multiple stroke centers to prospectively enroll consecutive patients with RSSI. The external validation cohort will be stratified by age, sex, and lesion location to ensure representativeness, and the predictive performance of the nomogram will be evaluated using calibration plots, discrimination metrics, and decision curve analysis. Second, our swallowing assessments did not differentiate between dysphagia subtypes or severity levels, which might obscure nuanced clinical correlations. Third, advanced imaging techniques, such as diffusion tensor imaging or functional MRI, were not employed, which could have provided further insight into the neural mechanisms underlying dysphagia in RSSI. Fourth, other radiological signs of CSVD, such as brain atrophy, cortical thickness, or microstructural alterations, were not assessed in this study. These features may also influence the risk of PSD and warrant consideration in future research.

## Conclusions

In summary, our study highlights that among the imaging markers of CSVD, the presence of CMBs and moderate to severe EPVS are significantly associated with the development of PSD in patients with RSSI. Additionally, we identified several independent predictors of PSD, including elevated hs-CRP levels, higher baseline NIHSS scores, increased total CSVD burden, and specific lesion regions. These variables were incorporated into a robust and clinically applicable nomogram model with excellent discriminatory ability and predictive accuracy. This predictive tool offers meaningful clinical utility by facilitating the early identification of patients at high risk for dysphagia, thereby supporting timely and personalized intervention strategies aimed at improving patient outcomes.

## Data Availability

Data will be made available on request.
